# Electroacupuncture Reverses CUMS-Induced Depression-Like Behaviors and LTP Impairment in Hippocampus by Downregulating NR2B and CaMK II Expression

**DOI:** 10.1155/2021/9639131

**Published:** 2021-11-12

**Authors:** Shuo Jiang, Zui Shen, Wenlin Xu

**Affiliations:** ^1^The First Affiliated Hospital of Zhejiang University of Traditional Chinese Medicine, Hangzhou 310006, China; ^2^Zhejiang University of Traditional Chinese Medicine, Hangzhou 310053, China

## Abstract

**Objective:**

Depression is a global mental health problem with high disability rate, which brings a huge disease burden to the world. Electroacupuncture (EA) has been shown to be an effective method for the treatment of depression. However, the mechanism underling the antidepressant effect of EA has not been clearly clarified. The change of synaptic plasticity is the focus in the study of antidepressant mechanism. This study will observe the effect of EA on LTP of hippocampal synaptic plasticity and explore its possible mechanism.

**Methods:**

The depression-like behavior rat model was established by chronic unpredictable mild stress (CUMS). EA stimulation (Hegu and Taichong) was used to treat the depressed rats. The depression-like behavior of rats was tested by weight measurement, open field test, depression preference test, and novelty suppressed feeding test. Long-term potentiation (LTP) was recorded at CA1 synapses in hippocampal slices by electrophysiological method. N-methyl-D-aspartate receptor subunit 2B (NR2B) and calmodulin-dependent protein kinase II (CaMK II) protein levels were examined by using western blot.

**Results:**

After the establishment of CUMS-induced depression model, the weight gain rate, sucrose preference rate, line crossing number, and rearing times of rats decreased, and feeding time increased. At the same time, the LTP in hippocampus was impaired, and the expressions of NR2B and CaMK II were upregulated. After EA treatment, the depression-like behavior of rats was improved, the impairment of LTP was reversed, and the expression levels of NR2B and CaMK II protein were downregulated.

**Conclusion:**

EA can ameliorate depression-like behaviors by restoring LTP induction, downregulating NR2B and CaMK II expression in CUMS model rats, which might be part of the mechanism of EA antidepressant.

## 1. Introduction

Depression is a major global mental health problem with high prevalence, recurrence rate, disability rate, and suicide risk. According to the WHO statistics, about 350 million people worldwide suffer from depression [1]. At present, depression has become the main cause of disability in the world and the second largest disease burden after ischemic heart disease [2,3]. The pathogenesis of depression is complex and unclear. Due to the lack of accurate and effective treatment targets, the curative effect of modern medicine in the treatment of depression is poor [4].

At present, the pathogenesis of depression is diverse, such as the regulation of monoamine neurotransmitters and their receptors in the brain, neuroendocrine disorders, neurotrophin malnutrition and nerve regeneration, changes in hippocampal neurons, cell signal transduction, and immune function [5]. In recent years, neural plasticity has gradually become a research hotspot [6–9]. More and more evidence shows that the regional specific changes of synaptic morphology and function are the result of chronic stress and depression. As the embodiment of system reorganization, neural plasticity continues through the normal development, maturation, and degradation of the nervous system. In order to understand the essence of neurological or mental diseases, neuroplasticity is an important aspect that cannot be ignored [10, 11].

Long-term potentiation (LTP) is a form of synaptic plasticity. The intensity of LTP is related to synaptic activity [12, 13]. LTP is a phenomenon such that the intensity of synaptic response increases for a long time after short series of high-frequency stimulation. LTP is generally considered to be the physiological basis of brain learning and long-term memory processes [14]. In recent years, scholars have found that hippocampal LTP abnormalities not only affect learning and memory functions, but also may be related to the occurrence of psychological and behavioral abnormalities such as anxiety and depression [15]. When a large amount of Ca^2+^ flows into the postsynaptic membrane from cells, LTP can be induced and can last for hours to days [16]. Compared with healthy people, LTP plasticity in patients with depression was significantly impaired. This attenuation of synaptic plasticity is restored after remission of depressive state [17]. The impaired LTP plasticity was a potential pathomechanism and treatment target of depression.

The most critical factor in the initiation of synaptic plasticity is calcium (Ca^2+^) influx through NMDA receptors [18]. Calcium influx triggers the activation of calcium/calmodulin-dependent protein kinase II (CaMK II) [19]. CaMK II can be regarded as an indicator of intracellular calcium level [20]. It is worth noting that when excess glutamate binds to NR2B, the overactivated extrasynaptic NR2B (inducing glutamate excitotoxicity) has strong calcium permeability [21]. In previous studies, we found that chronic unpredictable mild stress (CUMS) can lead to the overactivation of postsynaptic glutamate NMDA pathway in rat hippocampal astrocytes, resulting in the accumulation of glutamate and a large amount of Ca^2+^ flowing into neurons and astrocytes, resulting in intracellular calcium overload. NMDA receptor subunit NR2B plays an important role [22]. In CUMS-induced depressive rats, with the gradual aggravation of depressive symptoms, the expression of NR2B increased significantly and the content of intracellular calcium increased.

Electroacupuncture has a good antidepressant effect [23–25]. The mechanisms of electroacupuncture antidepressant include regulating neuropeptides and neurotransmitters [26], inhibiting HPA axis hyperactivity and inflammation [27], and restoring hippocampal synaptic plasticity [28]. Synaptic plasticity is the focus of electroacupuncture antidepressant research. Whether the effect of electroacupuncture on synaptic plasticity is related to NR2B is unclear. In this study, we explored the antidepressant effect of electroacupuncture and its possible mechanism by studying the effects of EA on hippocampal synaptic plasticity and NR2B protein level in CUMS rat models.

## 2. Materials and Methods

### 2.1. Animals

Healthy adult female Sprague Dawley (SD) rats (7-8 weeks old, weighing 220-250 g) were used in the experiment. All animals were obtained from Shanghai Experimental Animal Center, Chinese Academy of Sciences. Before further experiments, the rats were housed under a new environment (room temperature: 24 ± 1°C; relative humidity: 45 ± 15%; 12/12 h light/dark cycle) for one week. Study procedures were conducted according to the National Institutes of Health Guide for the Care and Use of Laboratory Animals. The research program and animal care followed the Committee's guidelines. 45 rats were randomly divided into three groups: control group (*n* = 15), CUMS group (*n* = 15), and CUMS + EA group (*n* = 15).

### 2.2. Chronic Unpredictable Mild Stress

**Figure 1:**
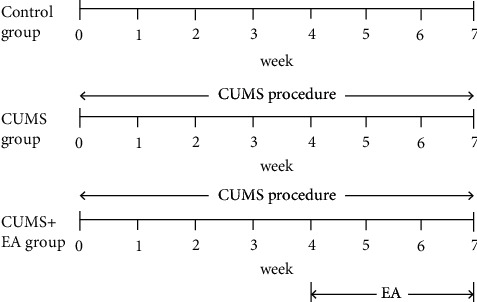
Experimental procedure.

Rats with depression-like behaviors were prepared by exposing the rats to chronic unpredictable mild stress [22]. The animals were isolated in separate cages and subjected to various stressors for 4 weeks. The stressors used in this study were water deprivation (24 hours), food deprivation (24 hours), tail pinch (1 minute), cold swim (4°C, 5 minutes), thermal stimulation (45°C, 5 minutes), foot shock (50 mV shock every 10 seconds for 30 seconds, a total of 15 shocks), and horizontal shaking (10 minutes). The animals were given a random stimulus at a random time every day and the same stressor was not applied consecutively over two days to ensure that the timing and manner of the stimulus occurrence were unpredictable. Rats in the CUMS group and CUMS + EA group were exposed to CUMS when rats in the control group received no other intervention except normal feeding (Figure 1).

### 2.3. Interventions

Rats in the CUMS + EA group received EA treatment after the establishment of rat depression model, while rats in CUMS group and the control group received grasping without other intervention (Figure 1). The rats were treated with electroacupuncture at the Siguan points including LI4 (Hegu) and LR3 (Taichong) on both sides for 3 weeks [24,29]. LI4 is located at the radial midpoint of the second metacarpal, and LR3 is located in the depression anterior to the junction of the first and second metatarsal.

Stainless steel needles of 0.18 mm diameter and 15 mm length (Suzhou Medical Appliance Factory, Suzhou, China) were inserted to a depth of approximately 2 mm of 4 acupuncture points. The electrical stimulation apparatus G6805-II (Qingdao Xinsheng Industrial Co., Ltd., Qingdao, China) was connected to needles at a 15 Hz continuous wave for 30 min each time. EA was performed once a day for the fifth week and once every other day for the sixth and seventh week.

### 2.4. Behavioral Tests

Behavioral tests were used to evaluate the establishment of the model and the efficacy of the antidepressant effects, including weight measurements, the open field test, the novelty suppressed feeding test, and the sucrose preference test. Record every week (days 0, 7, 14, 21, 28, 35, 42, and 49) to observe the physiological state during CUMS modeling.

#### 2.4.1. Weight Measurement

The weight of the rats in each group was measured every week and the weight gain rate of the rats was calculated as follows: (weight increase ratio = (body weight at measurement - body weight on the first day)/body weight on the first day ×100%).

#### 2.4.2. Open Field Test (OFT)

The apparatus was an 80 cm × 80 cm × 40 cm square wooden box, with black all around and covering the bottom. The box was placed in a soundproof room. The floor of the box was separated by white lines into 25 squares of equal size (each 16 cm × 16 cm).

Each animal was placed in the center of the box bottom, and a camera was used to observe and record the number of line crossings and number of rearings. After the behavior experiment of one rat, the feces and other residues in the box were cleaned immediately, and an alcohol cotton ball (75%) was used to wipe away the odor left by the previous rat to avoid interfering with the behavior of the next rat. Scoring: ① the number of line crossings: when more than three claws stepped into one square or the center of gravity fell into one square, the score was 1; ② number of rearings: when the forelimb left the horizontal ground, the score was 1.

#### 2.4.3. Sucrose Preference Test

① Preparation stage: two identical water bottles were placed in each cage at the same time. In the first 24 hours, two bottles of the same weight containing 1% sucrose water were used; in the second 24 hours, one bottle of 1% sucrose water and the other bottle of pure water of the same weight were used. Then, fasting and water prohibition were carried out for 24 hours. ② Experimental stage: each rat was given a bottle of 1% sucrose water and a bottle of purified water at the same time. After 24 hours, the consumption amounts of sucrose water and purified water were measured, and the preference rate of sucrose water was calculated as follows: (preference rate of sucrose water = consumption of sucrose water/(consumption of purified water + consumption of sugar water) ×100%).

#### 2.4.4. Novelty Suppressed Feeding Test

After 24 hours of fasting, rats were placed in an open field made of plexiglass (76.5 × 76.5 × 40 cm). A small amount of food was placed in the center of the open field. During the experiment, the rats were magnified from any corner into the open field, and each rat was placed in the open field for 6 minutes. The whole process was recorded on a computer by a video camera above the open field. The time of the first bite was recorded to the exact second. If the rat did not eat the food in 6 minutes, the result was recorded as no bite.

### 2.5. Western Blot

At the end of the seven weeks, rats were anesthetized by intraperitoneal injection with sodium pentobarbital (40 mg/kg) and decapitated. The hippocampi were homogenized in ice-cold RIPA lysis buffer and were centrifuged. The supernatant was resolved and separated by sodium dodecyl sulfate-polyacrylamide gel electrophoresis (10% gel) and transferred onto polyvinylidene fluoride (PVDF) membranes. Next, the membranes were blocked with 5% skimmed milk at room temperature for two hours and incubated overnight at 4 °C with primary antibodies at a dilution of mouse anti-CaMK II 1 : 2000 (Affbiotech, Changzhou, China). After washing with phosphate-buffered solution, the membranes were incubated with the horseradish peroxidase-conjugated goat anti-mouse immunoglobulin G secondary antibody 1 : 2000 (SA00001-15, Protein-tech, USA) for one hour, followed by extensive washing. Proteins were analyzed with an enhanced chemiluminescence system (ECL). Optical densities were measured by ImageJ software (National Institutes of Health, Bethesda, MD, USA). GAPDH was used as the loading control.

### 2.6. Immunofluorescence

Brain tissues were cut into 35 *µ*m thick slices until the entire structure of the hippocampus was detected. The slices were washed three times with 0.1 M phosphate-buffered saline (PBS) and incubated at room temperature for 20 minutes in 5% bovine serum albumin. Primary (overnight, 4°C) and secondary antibody incubations (one hour, room temperature) were carried out. The slices were washed three times with PBS after each incubation. Primary antibody was mouse anti-NR2B 1 : 200 (ab93610, Abcam). Secondary antibody was goat anti-mouse immunoglobulin G (Alexa Fluor 488) 1 : 2000 (ab150157, Abcam). Slices were coverslipped with a water-based mounting medium containing 4′,6-diamidino-2-phenylindole (DAPI) (KeyGEN BioTECH, Jiangsu, China). Stained sections were then observed by fluorescence microscopy (Olympus) and processed with ImageJ Software.

### 2.7. Long-Term Potentiation

At the end of the seven weeks, rats were anesthetized by intraperitoneal injection with sodium pentobarbital (40 mg/kg). After skin preparation, the rats were fixed on a stereotactic apparatus, then the hippocampus was drilled into them according to the coordinates (the position of stimulating electrode, P: -4.2 mm, R: 3.8 mm, and the position of recording electrode, P: -3.4 mm, R: 2.5 mm). The concentric circle stimulating electrode and metal recording electrode were gently inserted into the sites (Schaffer and CA1) of the hippocampus. The tip of the stimulating electrode was located in the hippocampal Schaffer collateral branches, and the tip of the recording electrode was located in the radiation layer of the CA1 area (located in hippocampal gyrus). When approaching the predetermined position, the insertion depth of the stimulating electrode and recording electrode in the subcortical area was slowly and precisely adjusted. At the same time, a direct current test stimulation with a width of 100 *μ*S and an intensity of 150 *μ*A was given every 10 seconds until the best field excitatory postsynaptic response (fEPSP) was reached. Then, the position of the electrode was fixed, the stimulation intensity of the basic fEPSP was recorded at 30–40% of the maximum response, and the electrode was recorded for 20 minutes to ensure the stability of the basic synaptic transmission. The high-frequency stimulation (HFS) to induce LTP was 200 Hz. Each stimulation consisted of 20 pulses with a total of 3 stimulation strings and an interval of 30 s. LTP was induced by HFS and recorded for 60 minutes to observe the LTP characteristics. The recorded signals were collected and amplified with an MP150 16-channel multichannel electrophysiological signal acquisition and processing system and displayed and stored on a multimedia computer. The experimental data and images were processed by SigmaPlot software.

### 2.8. Statistics

Statistical Product and Service Solutions (SPSS) 24.0 was adopted for data analysis. The results are presented as the mean ± standard error of the mean (SEM). Data normality was assessed by the Kolmogorov-Smirnov test. For statistical analyses of the behavioral tests within each group, a paired *t*-test was employed. One-way ANOVA and Tukey–Kramer's post hoc test were used for LTP statistical analysis. A *P* value < 0.05 was considered statistically significant.

## 3. Results

### 3.1. Behavioral Tests

**Figure 2:**
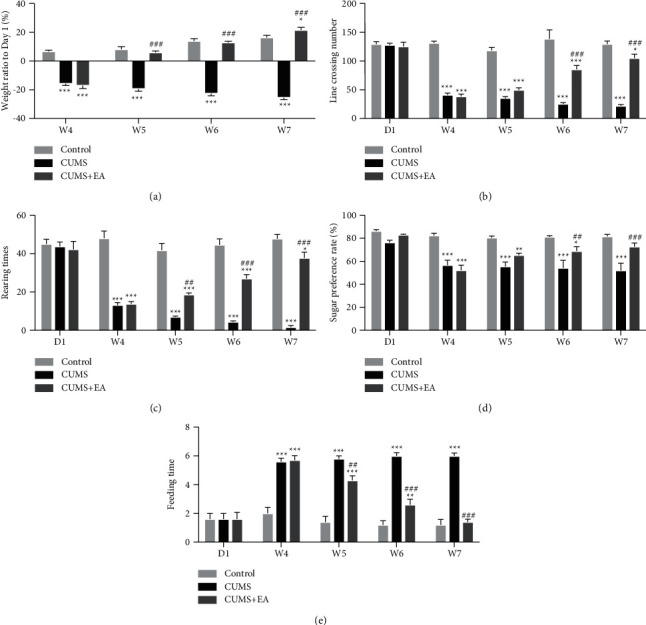
The effects of CUMS on body weight and behavior. (a) Body weight increase ratio of each group of rats at different time points (*W*4 = week 4, *W*5 = week 5, *W*6 = week 6, and *W*7 = week 7) relative to the ratio of the control group (^*∗*^*P* < 0.05, ^*∗∗∗*^*P* < 0.001) and to the CUMS group (^###^*P* < 0.001). (b) Number of line crossings. (c) Number of rearings. (d) Sucrose preference rate. (e) Time to first bite for each group at different time points relative to the control group (^*∗*^*P* < 0.05, ^*∗∗*^*P* < 0.01, and ^*∗∗∗*^*P* < 0.001) and to the CUMS group (^##^*P* < 0.01 and ^###^*P* < 0.001). Data are presented as the mean ± SEM, *n* = 15 for each group.

After modeling (on week 4), compared to the control group, the weight increase ratio of the rats in CUMS group and CUMS + EA group decreased significantly (^*∗∗∗*^*P* < 0.001, Figure 2(a)). During the treatment stage (from week 5 to week 7), the weight increase ratio of the rats in CUMS group decreased constantly (compared to the control group, ^*∗∗∗*^*P* < 0.001; compared to the CUMS + EA group, ^###^*P* < 0.001, Figure 2(a)). In contrast, this ratio in the rats in the CUMS + EA group increased constantly (compared to the CUMS group, ^###^*P* < 0.001, Figure 2(a)). At week 7, there was a significant difference between the control group and the CUMS + EA group (^*∗*^*P* < 0.05, Figure 2(a)). The weight increase ratios of the rats in the CUMS + EA group were higher than that in the control group.

The line crossing number was not significantly different between groups on day 1. After modeling, the number of line crossings of rats in CUMS group and CUMS + EA group significantly decreased. In weeks 4, 5, and 6, compared to the control group, the number of line crossings of rats in the CUMS group and CUMS + EA group decreased significantly (^*∗∗∗*^*P* < 0.001, Figure 2(b)). In week 6, compared to the CUMS group, the number of line crossings of the rats in the CUMS + EA group began to increase gradually (^###^*P* < 0.001, Figure 2(b)). In week 7, the number of line crossings of the rats in CUMS + EA group increased constantly (compared to control group, ^*∗*^*P* < 0.05; compared to CUMS group, ^###^*P* < 0.001, Figure 2(b)).

The increase in the number of rearing was not significantly different between each of the groups on day 1. From week 4 to week 7, there were significant differences between groups (^*∗*^*P* < 0.05, ^*∗∗∗*^*P* < 0.001, ^##^*P* < 0.01, ^###^*P* < 0.001, Figure 2(c)). The rising number had a gradual increase from week 5 to week 7 in the CUMS + EA group (compared to the CUMS group, ^##^*P* < 0.01, ^###^*P* < 0.001, Figure 2(c)). There was a significant decrease in the CUMS group throughout the whole experiment.

The sucrose preference ratio was not significantly different between each of the groups on day 1. From week 4 to week 6, compared to the control group, there were significant decreases in sucrose preference in the CUMS group and CUMS + EA group (^*∗*^*P* < 0.05, ^*∗∗*^*P* < 0.01, ^*∗∗∗*^*P* < 0.001, Figure 2(d)). On week 6, the sucrose preference ratio of the rats in the CUMS + EA group began to increase gradually (compared to the CUMS group, ^##^*P* < 0.01, Figure 2(d)). In week 7, the sucrose preference ratio of the rats in the CUMS + EA group increased constantly. There was no difference between the control group and the CUMS + EA group.

The time to first bite was not significantly different between each of the groups on day 1. Compared to the control group, the time to first bite of the rats in the CUMS group and CUMS + EA group increased significantly from week 4 to week 6 (^*∗∗*^*P* < 0.01, ^*∗∗∗*^*P* < 0.001, Figure 2(e)). Compared to the CUMS group, there was a gradual recovery from the rats in the CUMS + EA group from week 5 to week 7 (^##^*P* < 0.01, ^###^*P* < 0.01, Figure 2(e)). There was no difference between the control group and the CUMS + EA group at week 7. The time to first bite of the rats in the CUMS group was at a high level throughout the whole experiment.

### 3.2. Western Blot Results

**Figure 3:**
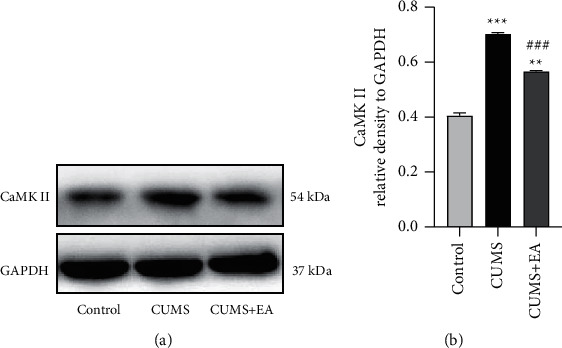
Expression of CaMK II in each group. (a) Western blot analyses of CaMK II protein levels in hippocampal homogenates. (b) Protein expression ratios relative to GAPDH. Data are given as the mean ± SEM, *n* = 5 for each group. ^*∗∗*^*P* < 0.01 and ^*∗∗∗*^*P* < 0.001 compared to the control group. ###*P* < 0.001 versus the CUMS group.

Protein expression of CaMK II was upregulated after modeling. Compared with the control group, the relative densities of CaMK II in the CUMS group and CUMS + EA group both increased significantly (^*∗∗*^*P* < 0.01 and ^*∗∗∗*^*P* < 0.001, Figure 3(b)). After EA treatment, the relative density of CaMK II decreased. There was a significant difference between the CUMS group and the CUMS + EA group (^###^*P* < 0.001, Figure 3(b)).

### 3.3. Immunofluorescence Results

**Figure 4:**
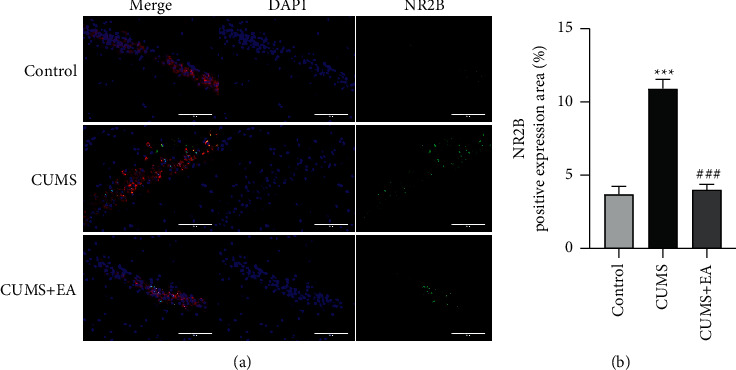
Expression of NR2B in each group. (a) Double-immunofluorescence micrographs showing NR2B-positive cells in the hippocampus of each group. (b) Positive expression area (%) of NR2B. Mean ± SEM, *n* = 5 for each group. ^*∗∗∗*^*P* < 0.001 compared to the control group. ###*P* < 0.001 compared to the CUMS group.

Compared to the control group, the expression of NR2B in cells increased significantly in the CUMS group (^*∗∗∗*^*P* < 0.001, Figure 4(b)). There was no difference in the expression of NR2B in the hippocampal cells between the control group and the CUMS + EA group. Compared to the CUMS group, the expression of NR2B in hippocampal cells decreased significantly in the CUMS + EA group (^###^*P* < 0.001, Figure 4(b)).

The immunofluorescence results showed that the expression of NR2B in the hippocampi of depressed rats was significantly upregulated. After EA treatment, the expression of NR2B decreased significantly.

### 3.4. Long-Term Potentiation Results

**Figure 5:**
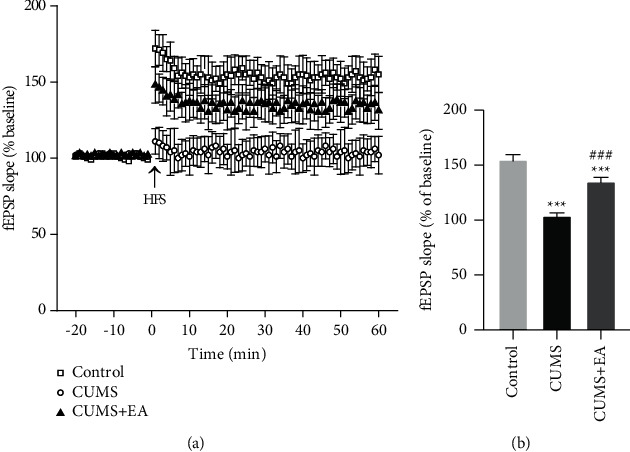
Induction of LTP in the hippocampal Schaffer collateral-CA1 in each group. (a) Time series plot of LTP in the three groups of rats. (b) Summarized data of the average slope change (normalized to the baseline value) from the three groups of rats. Means are shown and the SEM is displayed as error bars; *n* = 5 per group. ^*∗∗∗*^*P* < 0.001 compared to the control group. ###*P* < 0.001 compared to the CUMS group. One-way ANOVA with Tukey–Kramer post hoc test.

We recorded evoked fEPSP in the stratum radiatum of the CA1 region in rats in the control group, CUMS group, and CUMS + EA group. High-frequency stimulation (HFS) induced stable reaction of LTP in high level in control rats and depression-like rats with EA. High-frequency stimulation (HFS) induced a weak reaction of LTP in depression-like rats. As time went on, the LTP of depression-like rats kept in a lower level than the rats in the other two groups. LTP was significantly restricted in depression-like rats (Figure 5(a)). Compared to the rats in the other two groups, the increase of fEPSP in rats' hippocampi in CUMS group was not obvious under the induction of short string high-frequency stimulation (HFS).

Figure 5(b) summarizes the change of fEPSP slope for three groups. There was a great difference between CUMS model rats and the other two groups (^*∗∗∗*^*P* < 0.001, ^###^*P* < 0.001, Figure 5(b)). Compared to the control group, changes of fEPSP slope in CUMS group were smaller. Compared to CUMS group, the fEPSP slope in CUMS + EA group was significantly increased.

## 4. Discussion

In our former study, depression behavior (decreasing the weight gain rate, sucrose preference rate, line crossing number, and rearing times and increasing feeding time) was produced in CUMS-induced depressive rats. In our current study, the LTP in hippocampus was impaired, and the expressions of NR2B and CaMK II were upregulated in the CUMS-induced depression rats. After EA treatment, the depression-like behavior of rats was improved, and the change of LTP and the expression of NR2B and CaMK II were reversed. The results suggest that EA can improve depression-like behavior in model rats, which may be related to repairing LTP and downregulating NR2B and CaMK II.

Depression is a serious neurophysiological disorder with a complicated pathogenesis [5]. Most patients experience chronic stress for a long period of time. Some studies have shown that stressful life events, especially chronic agnostic stress events, are considered to be obvious predisposing factors of depression [30,31]. Chronic long-term stress has shown a dose-response relationship with depression. The more stressful life events there are, the higher the incidence rate of depression is, and the more severe the symptoms are [32]. Our team used CUMS to simulate the difficulties people encounter in real life, which is internationally recognized as an effective animal modeling method for depression [33]. The results showed that, with the extension of modeling time, compared with the control group, rats in the model group and treatment group showed decreases in their weight increase ratio, sucrose preference ratio, number of line crossings, and number of rearings. Moreover, the time to first bite in the open field of rats in the control group was reduced compared with that of the rats in the model and treatment groups.

Neurobiological studies have confirmed that the occurrence of depression is related to multiple brain regions [34,35], mainly the prefrontal cortex (PFC), anterior cingulate cortex (ACC), thalamus, hippocampus, amygdala, and basal ganglia. Damage to the hippocampus is considered to be closely related to depression, posttraumatic stress disorder, and other mental diseases. The hippocampus is an important target for the study of excitatory-dependent synaptic plasticity in the mammalian brain [36]. Therefore, the current study of synaptic plasticity mainly focused on the hippocampus [37,38]. In this research, we also focused on the hippocampus to study the relationship between depression, synaptic plasticity, and EA treatment.

The antineuroplasticity changes in depression include decreased proliferation of neural stem cells, decreased survival of neuroblasts and immature neurons, damaged neural circuits, decreased neurotrophin levels, decreased spinal density, and dendritic retraction [39]. The impairment of LTP maintenance is mainly due to the dysfunction of protein synthesis and the formation of new neuronal structures [40]. Nerve spines, dendrites, and synapses are also missing in patients with depression or rodents [41], which provides a basis for LTP damage. In CUMS depressed rats, the LTP of CA3 - CA1 synapses induced by high-frequency stimulation decreased significantly, indicating that depression leads to LTP injury and cognitive decline [42]. Chronic stress can decrease the strength of this synapse and impairs LTP, while antidepressant treatment can reverse stress-induced changes [43]. This study shows that electroacupuncture can reverse LTP impairment in depressed rats. In the CUMS group, high-frequency stimulation failed to induce LTP, indicating that synaptic plasticity was impaired in CUMS-induced depression model rats. Compared with CUMS group, the fEPSP slope of CUMS + EA group was significantly increased and LTP was successfully induced, indicating that the damaged synaptic plasticity of depression rats after electroacupuncture treatment was restored.

LTP is a phenomenon such that the intensity of synaptic response increases for a long time after short series of high-frequency stimulation. When a large amount of Ca^2+^ flows into the postsynaptic membrane from cells, LTP can be induced for hours to days [16]. The occurrence of LTP requires the transport of intracellular calcium into the postsynaptic membrane, and a large number of Ca^2+^ receptor proteins are involved in the process of calcium transport [44], one of which is CaMK. CaMK is a protease that is highly expressed in brain tissue, especially in the hippocampus. It plays an important role in neural plasticity and memory formation. It is the most important Ca^2+^ receptor protein in neurons. Calcium influx triggers the activation of CaMK II [45]. CaMK II might be viewed as an indicator of intracellular calcium level [20]. There existed a close relationship between intracellular Ca^2+^ overload and cell death [46]. CaMK II plays an important role in synaptic plasticity, and it can connect with NMDARs.

Excitotoxicity induced by intracellular calcium overload caused by overexcitation of NMDA receptor is considered to be an important pathological mechanism of many nervous system diseases [47,48]. The most critical factor in the initiation of synaptic plasticity is Ca^2+^ influx through NMDA receptors [18]. NMDARs are known for their role in the induction of LTP [49]. Among the many subunits of NMDARs, NR2B is more important for inducing LTP. It is worth noting that when excess glutamate binds to NR2B, the overactivated extrasynaptic NR2B (inducing glutamate excitotoxicity) has strong calcium permeability [21]. The interaction between CaMK II and NR2B plays an important role in the formation of dendritic axons, the establishment of synaptic connections, and the formation and maturation of synapses. It is necessary for synaptic plasticity [50]. The inhibition function of LTP and apoptosis of hippocampal neural cells were reversed with the decrease of NR2B and CaMK II protein levels [51]. In the current study, the relative ratio of CaMK II increased by CUMS and was reversed by EA.

The results showed that CaMK II and NR2B protein overexpression and LTP inhibition occurred in CUMS-induced depression model rats. After acupuncture treatment, LTP inhibition was effectively alleviated, and the expression levels of CaMK II and NR2B proteins were downregulated. These results suggest that the depression-like behavior of CUMS-induced depression rats is accompanied by the overexpression of CaMK II and NR2B proteins in hippocampus and the inhibition of LTP. Acupuncture and moxibustion can improve the above situation. The antidepressant mechanism of acupuncture may be related to inhibiting the overexpression of CaMK II and NR2B proteins, inducing stable LTP and improving synaptic plasticity.

## 5. Conclusion

EA can ameliorate depression-like behaviors by restoring LTP induction, downregulating NR2B and CaMK II expression in CUMS model rats, which might be part of the mechanism of EA antidepressant.

## Data Availability

The raw data used to support the findings of this study are available from the corresponding author upon request.
